# Is simulation-based team training performed by personnel in accordance with the INACSL Standards of Best Practice: Simulation^SM^?—a qualitative interview study

**DOI:** 10.1186/s41077-021-00186-w

**Published:** 2021-09-26

**Authors:** Anne Strand Finstad, Randi Ballangrud, Ingunn Aase, Torben Wisborg, Luis Georg Romundstad, Conrad Arnfinn Bjørshol

**Affiliations:** 1 Department of Nurse Anesthetists, Division of Emergencies and Critical Care, Oslo University Hospital, Oslo, Norway; 2 SHARE - Centre for Resilience in Healthcare, Faculty of Health Sciences, University of Stavanger, Stavanger, Norway; 3 Department of Health Science, Faculty of Medicine and Health Sciences, Norwegian University of Science and Technology, Teknologivegen 22, 2815 Gjøvik, Norway; 4 Anaesthesia and Critical Care Research Group, Faculty of Health Sciences, University of Tromsø – the Arctic University of Norway, Tromsø, Norway; 5 Norwegian National Advisory Unit on Trauma, Division of Emergencies and Critical Care, Oslo University Hospital, Oslo, Norway; 6 Hammerfest Hospital, Department of Anaesthesiology and Intensive Care, Finnmark Health Trust, Hammerfest, Norway; 7 Department of Anaesthesia, Oslo University Hospital, Rikshospitalet, N-0424 Oslo, Norway; 8 The Regional Centre for Emergency Medical Research and Development (RAKOS), Stavanger University Hospital, Stavanger, Norway; 9 Department of Anaesthesiology and Intensive Care, Stavanger University Hospital, Stavanger, Norway; 10 Department of Clinical Medicine, University of Bergen, Bergen, Norway

**Keywords:** Anaesthesia, Simulation-based team training, Framework, Objectives, Facilitation, Debriefing, Evaluation

## Abstract

**Background:**

Anesthesia personnel was among the first to implement simulation and team training including non-technical skills (NTS) in the field of healthcare. Within anesthesia practice, NTS are critically important in preventing harmful undesirable events. To our best knowledge, there has been little documentation of the extent to which anesthesia personnel uses recommended frameworks like the Standards of Best Practice: Simulation^SM^ to guide simulation and thereby optimize learning. The aim of our study was to explore how anesthesia personnel in Norway conduct simulation-based team training (SBTT) with respect to outcomes and objectives, facilitation, debriefing, and participant evaluation.

**Methods:**

Individual qualitative interviews with healthcare professionals, with experience and responsible for SBTT in anesthesia, from 51 Norwegian public hospitals were conducted from August 2016 to October 2017. A qualitative deductive content analysis was performed.

**Results:**

The use of objectives and educated facilitators was common. All participants participated in debriefings, and almost all conducted evaluations, mainly formative. Preparedness, structure, and time available were pointed out as issues affecting SBTT.

**Conclusions:**

Anesthesia personnel’s SBTT in this study met the International Nursing Association for Clinical Simulation and Learning (INACSL) Standard of Best Practice: Simulation^SM^ framework to a certain extent with regard to objectives, facilitators’ education and skills, debriefing, and participant evaluation.

**Supplementary Information:**

The online version contains supplementary material available at 10.1186/s41077-021-00186-w.

## Background

Simulation-based team training (SBTT) gives healthcare professionals the opportunity to learn and practice in safe environments without the risk of patient injury [1, 2]. Simulation is defined as “A technique that creates a situation or environment to allow persons to experience a representation of a real event for the purpose of practice, learning, evaluation, testing, or to gain understanding of systems or human actions” [3]. Anesthesia personnel was among the first to implement simulation and team training including non-technical skills (NTS) in healthcare [1, 4]. It has been stated that anesthesia has much in common with aviation and the nuclear industry, sharing safety as its primary goal [5]. Aviation introduced the term NTS as part of safety-related behavior. NTS are defined as “the cognitive, social, and personal resource skills that complement technical skills and contribute to safe and efficient task performance” [6]. These skills often include situation awareness, decision-making, teamwork, leadership, and the management of stress and fatigue [7]. In 2012, an international expert group recommended NTS as one of five topics (technical skills, non-technical skills, system probing, assessment, and effectiveness) to focus on in simulation-based training for improving patient safety [8]. Within anesthesia the NTS are critically important in preventing undesirable events involving the surgical patient [9, 10]. Specialized team-training programs in different settings have been introduced to improve NTS, including task management, team working, situation awareness, and decision-making [5, 11, 12].

A systematic review and meta-analysis [13] showed that SBTT in anesthesia affects outcomes such as satisfaction, knowledge, skills, and behavior of anesthesia personnel. Several studies in anesthesia settings have shown that SBTT improves team performance, cultural attitudes and perceptions, and communication climate among anesthesiologists and obstetricians in teamwork [14]; technical skills and NTS during the management of malignant hyperthermia management [15]; NTS and clinical actions during weaning from cardiopulmonary bypass [16]; trauma team performance [17]; and resuscitation skills and team performance during neonatal resuscitation [18].

In recent years, standardized frameworks for simulation-based team training have been introduced [19, 20]. We are not aware of studies on the extent to which anesthesia personnel follows recommended frameworks in simulation to optimize learning outcomes. SBTT has been conducted for decades, but in 2016, a new standard of best practice was introduced.

The International Nursing Association for Clinical Simulation and Learning (INACSL) published Standards of Best Practice: Simulation^SM^ [20], an evidence-based framework to guide important areas in simulation. It reinforces simulation as a state-of-the-science teaching and learning strategy that may improve the conduct of simulations, learning outcomes, and compliance for clinical healthcare personnel. We chose the INACSL Standard as an evaluation tool for the simulation-based team training (SBTT) because it stands as an essential framework and a core proficiency of simulation education [21].

Concurrently with the advancement of simulation science, the standard is continuously evolved [20, 22, 23] and a guide simplifies the implementation [21].

For this study, we hypothesized that anesthesia personnel largely follow the INACSL 2016. The INACSL covers eight areas: design, outcomes and objectives, facilitation, debriefing, participant evaluation, professional integrity, simulation-enhanced interprofessional education, and a simulation glossary. Based on earlier research and theory [24–28], the four areas outcomes and objectives, facilitation, debriefing, and participant evaluation are the core areas in simulation and therefore selected for this study. The result of this study will show on a national level, which is unique, to which extent anesthesia personnel follows recommended frameworks in simulation in order to optimize learning outcomes and contribute to close this gap in the literature.

The aim of the study was to explore how anesthesia personnel in Norway conducts simulation-based team training (SBTT) of non-technical skills (NTS) with respect to four of these: outcome and objectives, facilitation, debriefing, and participant evaluation.

## Methods

### Design

We used a qualitative descriptive study design, based on individual interviews with one key person at each hospital, to explore their experience with SBTT and the four areas of the framework. The use of both closed and open-ended questions gave the participants the opportunity to illuminate the various facets of SBTT in a complete way [29].

### Sample and setting

Altogether, 54 Norwegian public hospitals were approached through simulation networks and other professional networks [30] and one participant from each hospital was selected based on his/her experience and responsibility for anesthesia personnel’s SBTT and answered the questions on behalf of them. The participants were nurse anesthetists, anesthesiologists, and registered nurses. A total of 51 public hospitals participated in the study. Two non-university hospitals and one affiliated with a university hospital chose not to participate. The participating hospitals represented different locations of SBTT (Table 1).

**Table 1:**
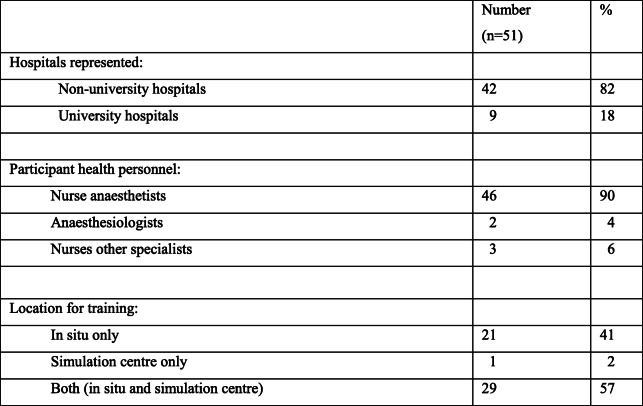
Descriptions of samples and settings

### Data collection

A semi-structured interview guide based on closed and open-ended questions (see Additional file 1) was prepared to address the aim of the study. The open-ended questions were specially designed to gather new knowledge [29]. Two pilot interviews were conducted to validate the interview guide; as a result, a question was added regarding the transfer of learning from simulation to clinical practice. The interview guide was sent to the participants in advance.

Data were collected by individual telephone interviews conducted by the first author (ASF). All participants were asked the same questions, and follow-up questions were used to encourage the participants to deepen or clarify their responses. The median interview length was 35 min (range 20–52 min). The first author conducted all the interviews from August 2016 to October 2017.

### Data analyses

A qualitative deductive content analysis based on Elo and Kyngäs [31] was used to deepen the understanding of the anesthesia personnel’s experiences with the conduct of SBTT. Data were analyzed according to the INACSL framework [20] focusing on the four areas; outcomes and objectives, facilitating, debriefing, and participant evaluation (see Additional file 2). The deductive analysis was organized according to three phases: preparation, organizing, and reporting [31]. In the preparation phase, the first author (ASF) transcribed the interviews and read through them several times to gain familiarity with the text and to understand the content and categorize the participants’ statements [32, 33]. The interviews were analyzed one by one. In the organizing phase, the authors (ASF, RB, CAB, and IA) established a structured analysis matrix designed in relation to the four areas [20]. The first author (ASF) reviewed the transcripts, the highlighted text was coded using the predetermined areas, and aspects that fit into the matrix were chosen (Table 2). The first author (ASF), with professional guidance from the three authors RB, CAB, and IA, completed the coding and analysis, together with viewpoints from TW and LGR. There were no discrepancies between the authors. In the reporting phase, the authors (ASF, RB, CAB, and IA) agreed on which citations to be used to supplement the text, to illustrate the four areas [31]. The analysis was done in original language and four authors (ASF, RB, CAB, and IA) approved the translation. The results are reported according to the COREQ Checklist [34] (Additional file 3).

**Table 2:**
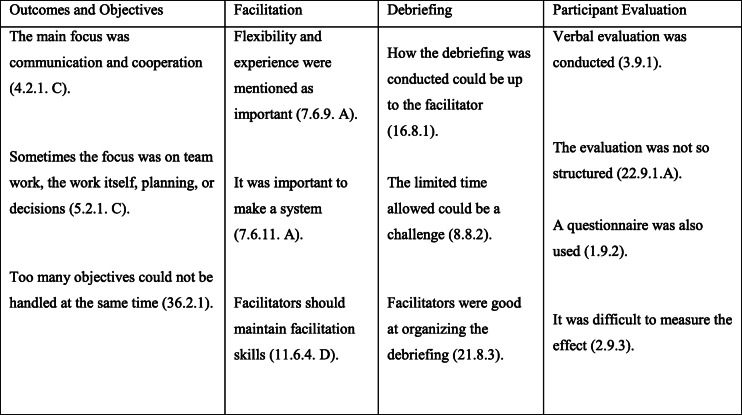
Codebook examples from the qualitative deductive content analysis

## Results

The summarized data based on the closed questions are presented in Table 3. A description of the qualitative data according to outcomes and objectives, facilitation, debriefing, and participant evaluation follows.

**Table 3:**
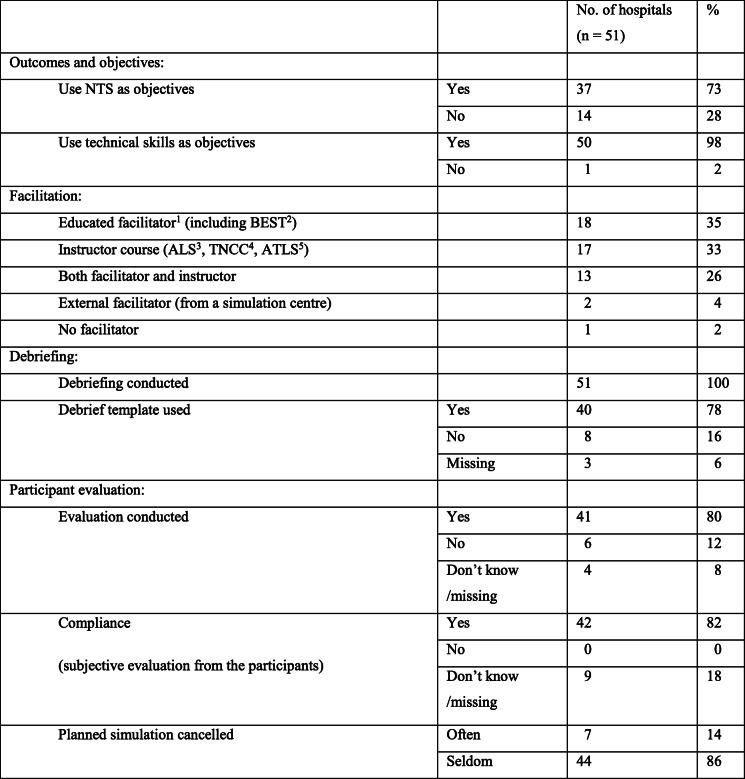
Summarized data based on the closed questions

^1^ Educated facilitators were defined as having participated in a course equivalent to the EuSim simulation instructor course level 1 [41], or been trained as a BEST instructor [42]

^2^*BEST* Better and Systematic Team Training, ^3^ *ALS* Advanced Life Support, ^4^*TNCC* Trauma Nursing Core Course, ^5^*ATLS* Advanced Trauma Life Support

### Outcomes and objectives

A total of 73 percent (*n*=37) of the participants used objectives including NTS (Table 3). They focused above all on teamwork and collaboration, as one said:

Distinctive communication—that is what it mostly boils down to… (No. 28)

Leadership, decision making, problem-solving, and situational awareness were also highlighted and 98% (*n*=50) had technical skills as objectives, for example, managing difficult airways. Their purpose was to enable the team to handle the situation to know what to do, where to acquire information from, where medical equipment is located, and who does what. As one mentioned:

…they should be able to act without panic… (No. 42)

Some were more concerned with the conduct of the scenario, but still discussed what to focus on:

It [the objectives] can be read between the lines, to put it in a way. (No. 43)

One mentioned that their colleagues wanted to achieve too many objectives in the same scenario:

It is like someone is too eager to train us in everything. I mean, when there are 30 training items, I don’t think it will be a good training. (No. 36)

The team members’ preparedness was highlighted as an important pedagogical aspect, and one of the success factors within the simulations:

… make it predictable, planned, provide information ahead of time and the exact information about what to practice, not exactly the setting, but all the information about the subject. (No. 12)

### Facilitation

The results showed that 61% (*n*=31) of the participants used educated facilitators. Some sent personnel to a facilitator course, and others invited external instructors to conduct these courses locally in the hospital. It was pointed out as a paradox that institutions would send personnel to expensive courses yet not have the capability to use this resource afterwards because, for example, the same personnel was too busy running the clinics.

Four percent (*n*=2) had support from a simulation center or a trauma center to make the pedagogical arrangements for the team training. Several mentioned the trauma-team training and treatment as structured and established, with experienced facilitators.

Elements and attitudes that were mentioned as important were the following:

…flexibility …experience…you can perform in spite of all distractions; …a system is incredibly important…you must manage to have the required patience. (No. 7)

### Debriefing

The result was 100% (*n*=51) conducted debriefings, and they regarded facilitators as essential, as one expressed:

Educated facilitators are good at going through those questions, following the template… (No. 21)

A total of 78% (*n*=40) used a debriefing template, but some simplified the content after a while. Others used objectives or guidelines from BEST or the Norwegian Resuscitation Council as a debriefing template. Just 16% (*n*=8) used no template. One put it this way:

…we try to do the debriefing in a way that reflects the objectives; sometimes we lose the thread, but we try to catch up on the initial plan… (No. 4)

Lack of time was a challenge. Some prioritized debriefing. Others conducted a very short debriefing standing in the corridor. Video recording was sometimes used to save time:

…then we don’t take the time to make the round, because everyone has watched it on the screen, so that is an advantage… (No. 7)

Team members were given the opportunity to talk before the video was played back. Some thought using video was too technically inconvenient. Observers and patient-actors gave valuable feedback and some used a specialist (e.g., a consultant) to make comments on medical issues. Team members were encouraged to describe their own views of the scenario.

### Participant evaluation

A total of 80% (*n*=41) completed a participant evaluation, which usually was formative and unstructured (often an oral conversation). Some used a formative structured evaluation (a report or a questionnaire). As one commented:

We ask the participants about their technical and non-technical skills before…and …after the course day…We see whether competence has developed during the day…Mostly we describe how well we think it has worked. (No. 1)

Observers could be useful in the evaluation process, but no structure or framework was reported. As much as 82 percent (n=42) said they could observe (subjectively) a connection between the simulation and behavior in a real situation afterwards; this could include more specific messages from team leaders and improved teamwork. Some expressed the following thoughts:

I saw that he [a simulation trainee] was very calm and very clear in what to do next and so on, so then I saw the effect… (No. 29); …when someone [a colleague] has been away from the hospital, comes back [after the training] and tells us…we haven’t seen this before, what has happened? (No. 12)

Participants also described their own experience:

I’m aware of it myself as well, that I perform better and know what my options are. (No. 46)

Feedback from other departments and professions was expressed like this:

…there has been positive feedback from other medical staff: air ambulance and hospitals we admit patients to. (No. 31) I know that air ambulance teams prefer to come to our hospital with seriously injured patients, because things work well. (No. 28)

## Discussion

**Fig. 1:**
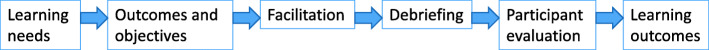
Four areas of INACSL that will facilitate the transformation of learning needs into learning outcomes

The aim of the study was to explore how anesthesia personnel in Norway conduct SBTT of NTS with respect to four areas [20]: outcomes and objectives, facilitation, debriefing, and participant evaluation. By following these recommendations, it is supposed to transform learning outcomes [20] (Fig. 1). All four topics were addressed, but to different degrees.

### Outcomes and objectives

Most participants reported the use of objectives, including NTS (Table 3). Nevertheless, 14 participants reported not using NTS as objectives, although some decided what to focus on. The INACSL framework recommends determining which objectives the participants should focus on in advance [20]. Not deciding outcomes and objectives in advance could result in failure to attain the intended quality and safety standards [20]. Despite the extensive use of objectives, improvement is needed in order to achieve the expected outcome for SBTT. Determining objectives in advance based on identified needs is recommended in the INACSL standard, as realism and fidelity alone do not necessarily produce more learning [1, 20]. In our view, the standard is of great importance in guiding facilitators and team members working with objectives to reach expected outcomes. Some of the objectives may not lead to improvement, as the real challenge could be something else (e.g., culture), which can be addressed using a process called system probing [8]. In situ simulation was common (Table 1) and revealed workplace-specific challenges (e.g., the location of equipment). Revealing these challenges could be crucial for clinical work and further SBTT, especially system probing.

Including too many objectives was considered problematic. Interdisciplinary collaboration where other professions want their own specific objectives in addition to the team-specific objectives is common. According to the INACSL, limiting the number of objectives is essential for success [20].

Participants also mentioned preparedness as a success factor. An interdisciplinary, unannounced in situ simulation study reported that 33% of participants experienced stress and unpleasantness [35], while a cardiac-arrest simulation study reported positive reactions from participants to unannounced in situ SBTT, as it better represented actual behavior [36]. No significant difference between unannounced and announced in situ simulations was reported in an emergency department [37]. One solution is that team members in SBTT could be informed about a planned simulation without being given information about when it will be performed. Realistic actual behavior could then be included as a training element [36].

Predictability and well-designed objectives based on needs seem to be success factors and crucial to achieve expected outcomes.

### Facilitation

A team member-centered facilitative approach is recommended, guided by the objectives, team members’ experience, and expected simulation training outcomes. Facilitators with formal training in simulation-based pedagogy are required to lead team members through SBTT [20], by giving instructions, feedback, and soliciting reflections, often called debriefing [25]. More than half the hospitals used educated facilitators. Some used external crew to support the local facilitator or instructor. Participants in our study reported frustration as the facilitators’ ordinary clinical work competed with the SBTT. Thus, implementation of simulation training with the intention of achieving expected outcomes requires both access to facilitators and additional clinical resources [20].

Participants pointed out trauma-team training as structured and established, with experienced instructors. A high frequency of this type of training could be a reason for this [38]. Facilitator experience is a prerequisite for flexibility and systematizing. To acquire sufficient experience and the recommended updating of their competence [20], the facilitators are dependent on managers’ priorities.

The use of a consistent facilitative approach to achieve intervention fidelity is recommended [20], and it is necessary to use skilled educators, for example, in the debriefing, to close performance gaps [1]. Participants in the study reported using a shortened and simplified debriefing template. This was explained by the limited time available, lack of updating, or infrequent simulation experience. SBTT with qualified facilitators is a way to achieve and maintain key competence among anesthesia personnel [1, 25].

### Debriefing

The intent of debriefing is to help team members to understand what they thought, felt, and did during the simulation and reflect on what knowledge to transfer into clinical practice to improve future performance [26]. Everyone in the study conducted debriefings. This was expected since almost all hospitals used educated facilitators or instructors who know that debriefing is an essential element in simulation [20, 26, 27, 39]. Debriefing should be congruent with outcomes and objectives [20], and some participants reported using objectives as a debriefing template, in line with the INACSL. A template (e.g., with descriptive, analytic, and reflective phases) is used in facilitator courses and used in the debriefing practice together with the objectives and outcomes. When the time was limited, some shortened the debriefing template. The consequences of this may be that fewer learning outcomes and behavioral changes are achieved and that the debriefing is perceived as deficient [20]. In order to successfully achieve the desired outcomes, it is crucial to use an experienced facilitator, who could prioritize important debriefing elements, especially when time is limited.

A video was mentioned as a time-saving tool as video playback replaced participants describing the event.

A systematic review showed that video-assisted debriefing has benefits comparable to verbal debriefing for learning outcomes, including experience, attitude, and performance, but not knowledge acquisition [40]. INACSL recommends using video if appropriate during feedback. The video has also been shown to improve clinical performance when used in clinical debriefing [18]. In our results, most users of video used verbal debriefing, followed by a video presentation to illustrate important elements. However, it is important to avoid the technical inconvenience that disturbs concentrated attention during debriefing.

### Participant evaluation

Most participants conducted a formative evaluation, such as an oral conversation, to develop the team members professionally and personally and reach the intended goals; however, very few used a summative evaluation, such as a questionnaire or rating scale, to measure the outcome of the single training.

Those omitting an evaluation could lose valuable support to individuals’ progress and the assessment of results and outcomes [20]. Educated facilitators should be aware of the recommended evaluation elements and prioritize them. Simulations led by uneducated facilitators can result in a lack of support for team members’ clinical competencies and further that gaps in knowledge and skills are not revealed [1, 20]. Those who did use structured evaluation with, e.g., a questionnaire could demonstrate that these issues were addressed during SBTT. Several observations of improved technical skills and NTS among anesthesia personnel were made. While observation frameworks were not mentioned, unstructured subjective observations regarding the SBTT were described. There is a need to document that SBTT results have a clinical impact [8]. The participants expressed the value of evaluating team members’ behavior, but the structured performance of this evaluation according to the INACSL seems to be lacking. Observations, individuals’ personal experiences, and feedback from other professionals showed the team members’ satisfaction with the SBTT and learning transformation in the study. Kirkpatrick described four levels of learning: (1) reaction, (2) learning, (3) behavior, and (4) results [28]. In our study, the participants reported about levels 1 to 3. However, we received no reports on level 4.

This study has revealed that the four areas of INACSL are followed to varying degrees in anesthesia SBTT. By stricter adherence to these four areas of INACSL, which is continuously evolved [20, 22, 23], we believe that anesthesia personnel can improve the transformation of learning needs into learning outcomes (Fig. 1). The framework is comprehensive, but could provide an awakening in addition to simplifying the implementation [21].

### Limitations of the study

The study is limited to one country. The participants mainly consisted of nurse anesthetists. This is due to the hospitals’ selection of contacts; finding healthcare professionals with the most extensive experience and responsibility for anesthesia SBTT.

A greater proportion of anesthesiologists could have given the study a broader perspective. Some participants could have been influenced by their previous involvement in SBTT and could have had more than one perspective, for example, if they had been both a facilitator and a member of a clinical team using SBTT.

With a survey, we could have included more hospitals and countries in our study. However, we chose interviews instead of a survey, as interviews give us a deeper understanding of the responses.

Of eight INACSL 2016 areas, the four most relevant areas were chosen with respect to the aim of the study, and to limit the study volume.

### Future perspectives

Further research is needed to assess SBTT with respect to other frameworks. Future studies are needed to examine whether a stricter adherence to INACSL guidelines improves learning outcomes based on learning needs.

## Conclusion

SBTT for anesthesia personnel in Norway meets the INACSL Standard of Best Practice: Simulation^SM^ framework in relation to outcomes and objectives, facilitation, debriefing, and participant evaluation to a certain extent. NTS were the main objectives used and are important to achieve the aim of SBTT and thereby achieve simulation quality standards. More than half the hospitals used educated facilitators, but they needed more frequent simulation training. Everyone conducted debriefings, but an improved use of the template is necessary to achieve expected outcomes. Most accomplished participant evaluations, which could be more structured and summative. Further research is needed in order to document any improvement in clinical results following increased adherence to INACSL during SBTT in anesthesia.

## Supplementary Information

**Additional file 1.** Interview guide.

**Additional file 2.** INACSL frameworks – four areas.

**Additional file 3.** COREQ Checklist.

## Data Availability

The study data and material are not available to anyone other than the authors, due to the participants’ consent agreement and the confidentiality policy.
