# Supplementary material for: Is simulation-based team training performed by personnel in accordance with the INACSL Standards of Best Practice: SimulationSM?—a qualitative interview study
Source: Adv Simul (Lond). 2021 Sep 26;6:33. doi: 10.1186/s41077-021-00186-w (PMC8474884; doi:10.1186/s41077-021-00186-w)
Supplement: Supplementary file 1 — Additional file 1. Interview guide. [file 41077_2021_186_MOESM1_ESM.pdf]

Additional file 1: Interview guide (chosen questions relevant for this article)

Preliminary questions:	
	Kind of hospital?
	What is your profession and function?
Closed and open ended questions:	
	Do the simulation take place in-situ or in simulation centre or both?<br>• If so, is the simulation centre inside or outside the hospital?
	Who is responsible for the pedagogical arrangements and preparation of scenarios?
	How easy/difficult is it for the anaesthetic personnel to participate in simulation training?
	Are there much/few cancellations?<br>• Reason?
	Which elements in the simulation training do you have? /How is the training prepared?<br>According to:<br>• Team training<br>• Technical skill training<br>• Non-technical skill training<br>• Information/Briefing<br>• Scenario (number)<br>• Debrief<br>• Evaluation
	Which objectives do you have?<br>How do you present the objectives?
	Do you have your own facilitators or instructors?<br>• What kind of education do they have?
	Do you conduct debriefing?<br>• If so how?
	Do you measure the effect of the simulation training?
	Have you observed any compliance in the clinic?
