# Supplementary material for: Is simulation-based team training performed by personnel in accordance with the INACSL Standards of Best Practice: SimulationSM?—a qualitative interview study
Source: Adv Simul (Lond). 2021 Sep 26;6:33. doi: 10.1186/s41077-021-00186-w (PMC8474884; doi:10.1186/s41077-021-00186-w)
Supplement: Supplementary file 2 — Additional file 2. INACSL frameworks – four areas. [file 41077_2021_186_MOESM2_ESM.pdf]

Additional file 2:

INACSL Standards of Best Practice: Simulation^{SM} - Four areas

	**Criteria Necessary to Meet This Standard**
**Outcomes and Objectives**	• Determine expected outcomes for simulation-based activities and/or programs.<br>• Construct Specific, Measurable, Achievable, Realistic, Time-phased objectives based on expected outcomes.
**Facilitation**	• Effective facilitation requires a facilitator who has specific skills and knowledge in simulation pedagogy.<br>• The facilitative approach is appropriate to the level of learning, experience, and competency of the participants.<br>• Facilitation methods before the simulation-based experience include preparatory activities and a prebriefing to prepare participants for the simulation-based experience.<br>• Facilitation methods during a simulation-based experience involve the delivery of cues (predetermined and/or unplanned) aimed to assist participants in achieving expected outcomes.<br>• Facilitation after and beyond the simulation-based experience aims to support participants in achieving expected outcomes.
**Debriefing**	• The debrief is conducted in an environment that is conducive to learning and supports confidentiality, trust, open communication, self-analysis, feedback, and reflection.<br>• The debrief is facilitated by a person(s) who can devote enough concentrated attention during the simulation to effectively debrief the simulation-based experience.<br>• The debrief is based on a theoretical framework for debriefing that is structured in a purposeful way.<br>• The debrief is congruent with the objectives and outcomes of the simulation-based experience.
**Participant Evaluation**	• Determine the method of participant evaluation before the simulation-based experience.<br>• Simulation-based experiences may be selected for formative evaluation.<br>• Simulation-based experiences may be selected for summative evaluation.<br>• Simulation-based experiences may be selected for high-stakes evaluation.

INACSL, *INACSL Standards of Best Practice: SimulationSM.* ELSEVIER, 2016.
